# A major sea-level drop briefly precedes the Toarcian oceanic anoxic event: implication for Early Jurassic climate and carbon cycle

**DOI:** 10.1038/s41598-019-48956-x

**Published:** 2019-08-29

**Authors:** François-Nicolas Krencker, Sofie Lindström, Stéphane Bodin

**Affiliations:** 1 Department of Geoscience, Aarhus University, Høegh-Guldbergs Gade 2, 8000 Aarhus C, Denmark; 2 GEUS–Geological Survey of Denmark and Greenland, Øster Voldgade 10, DK-1350 Copenhagen K, Denmark

**Keywords:** Palaeoclimate, Sedimentology

## Abstract

Sea-level change is an important parameter controlling the expansion of oxygen-depleted conditions in neritic settings during oceanic anoxic events (OAEs). Despite this fundamental role, it remains on a short timescale (<1 Myr) one of the least constrained parameters for numerous OAEs. Here we present sedimentological and geochemical evidence from Morocco and East Greenland showing that a forced regression shortly precedes (ca.10^2^ kyr) the major transgression associated with the Toarcian OAE. The forced regression can be correlated over distances greater than 3000 km in numerous Tethyan and Boreal basins, indicating that the relative sea-level change was driven by eustastic fluctuations. The major amplitude (>50 m) and short duration of the forced regression suggests that it was most likely related to the transient waxing and waning of polar ice sheet. We suggest that this short-lived glaciation might have a genetic link with the inception of the Toarcian OAE. Indeed, during the deglaciation and the accompanying sea-level rise, the thawing permafrost may have released important quantities of methane into the atmosphere that would have contributed to the Toarcian OAE rapid warming and its characteristic negative carbon isotope excursion. This study offers a hypothesis on how some hyperthermal events might be rooted in short-lived “cold-snap” episodes.

## Introduction

The early Toarcian Oceanic Anoxic Event (T-OAE) was one of the major environmental perturbations occurring during the Mesozoic^1–4^. The T-OAE was associated with an important faunal and floral turnover^5–9^ as well as soaring global temperatures^3,10–12^ and increased tropical cyclone intensity^13^. The T-OAE is best characterized by a high amplitude negative carbon isotope excursion recorded in carbonate micrite, bulk organic matter, wood debris, brachiopod valves, biomarkers, and organic matrix of belemnite rostra^2,3,14–19^. This has been observed in both shallow- and deep-water settings^13,20,21^, widely distributed over several terranes^18,20,22–25^, underlining the global character of this carbon cycle perturbation. Generally, a causal link between the emplacement of the Karoo-Ferrar large igneous province and the initiation of the T-OAE is postulated due to the synchronicity of these two events^26–28^. However, the exact mechanism responsible for the faunal and floral turnover at the onset of the T-OAE remains uncertain^7,8,29,30^, as well as the exact causes of the negative carbon isotope excursion^1,18,19,31–33^.

Another poorly constrained parameter of early Toarcian environmental perturbations concerns eustatic sea-level change. The creation/destruction of shallow-marine habitats due to sea-level change is known to be a primary control of marine biotic diversity^7,34,35^. Despite this, and the equally important role of relative sea-level change in guiding oceanographic currents and the development of anoxic bottom water, there is currently no consensus about the amplitude and interpretation of early Toarcian high-frequency sea-level changes. It is commonly accepted that, following the latest Pliensbachian “Spinatum” lowstand^36–38^, the early Toarcian corresponds to a long-term transgression associated with a global sea-level rise^38^, initially invoked as a cause for basinal anoxia/euxinia during the T-OAE^39^. However, several studies have highlighted that a short-term regressive event characterizes the upper part of the Polymorphum Zone^29,40–45^. Nevertheless, it remains elusive if this was only a normal regression (i.e. progradation driven by sediment supply outpacing the rate of base-level rise at the coastline) or if it was coupled to a forced regression (i.e. progradation driven by base-level fall). Presently, the amplitude of the sea-level rise that contributed to the overall Toarcian transgression is unconstrained as well as its exact cause and role in the unfolding of the T-OAE.

Here, we present new sedimentological, paleontological, and geochemical evidence from the Central High Atlas Basin (Morocco) and Jameson Land (East Greenland) in order to highlight the occurrence of a major forced regression (in the order of 50 m of base-level fall) prior to the onset of the T-OAE, during the latest Polymorphum Zone. We then show through literature review that the Polymorphum regressive event can be correlated over much of the western Tethys and the Boreal Sea, indicating that it was driven by eustatic sea-level fluctuations. Finally, the causes and consequences of this forced regression are discussed in the context of the T-OAE.

## The Central High Atlas Basin, Morocco

**Figure 1:**
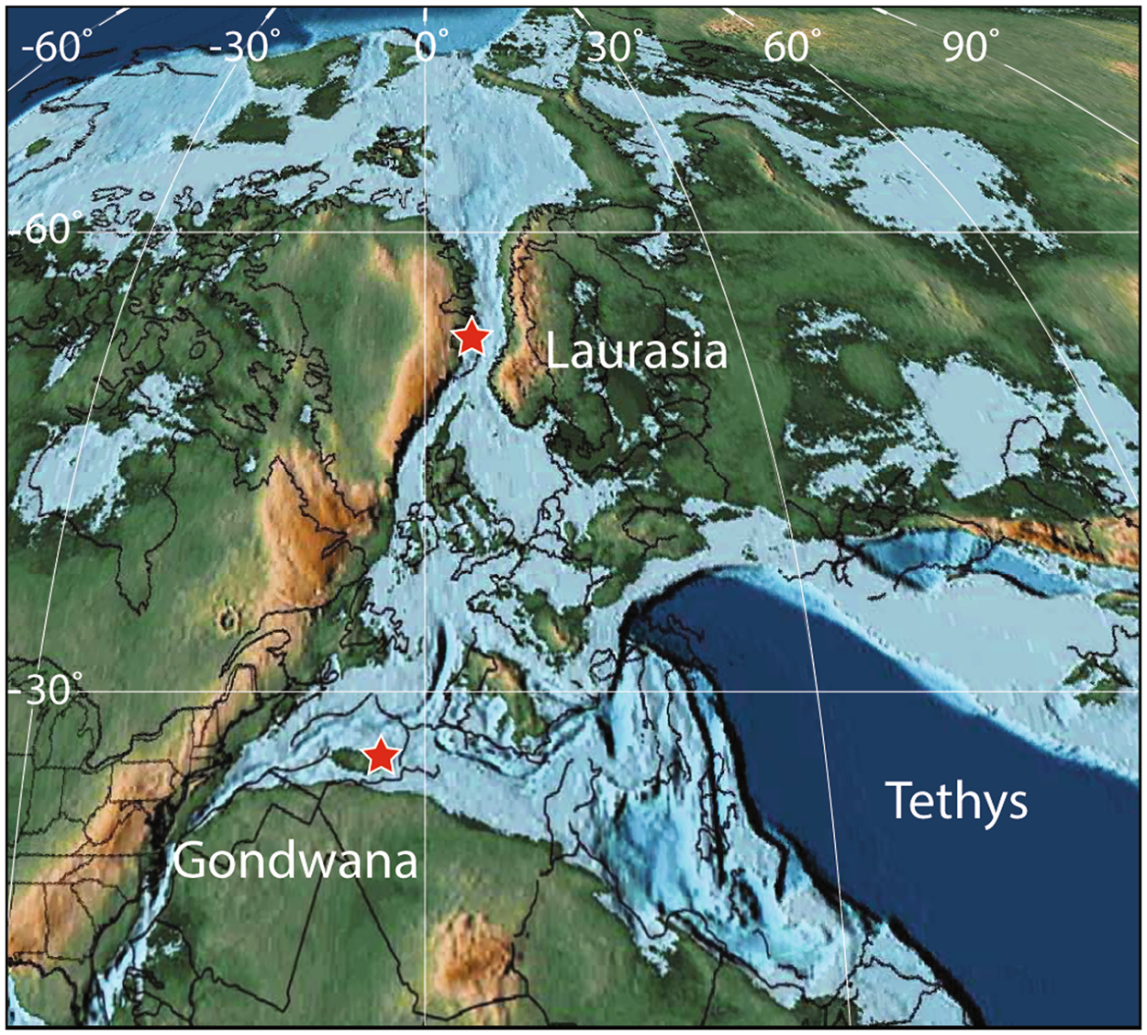
Early Jurassic paleogeographic map of the western Tethys (after ref.^96^) showing the location of the Central High Atlas Basin along the northern Gondwana margin and Jameson Land along the East Greenland margin. Maps generated with Adobe Illustrator CC 2015, http://www.adobe.com/products/illustrator.html.

The Central High Atlas Basin was located along the NW margin of the Tethys Ocean and its sedimentary sequences are currently exposed in the High Atlas Mountains (Fig. 1). It was characterized by a complex network of aborted asymmetric rift basins formed during the Late Permian–Triassic^46^. During the Early Jurassic, neritic sedimentation took place in the margins of the Central High Basin, which was opened towards the Tethys to the east. It was mostly dominated by biogenic carbonate deposition, but was interrupted twice by siliciclastic-dominated sedimentation during the early and late Toarcian. These siliciclastic pulses have been interpreted as reflecting climatic changes from dry to more humid conditions^11,18,47,48^. Within the southern Central High Atlas, two areas were studied: the Dades Valley and the surrounding of Amellago (Fig. 1). During the Toarcian, these localities were situated in shallow- and deep-neritic settings, respectively.

### Dades Valley

#### Stratigraphy

**Figure 2:**
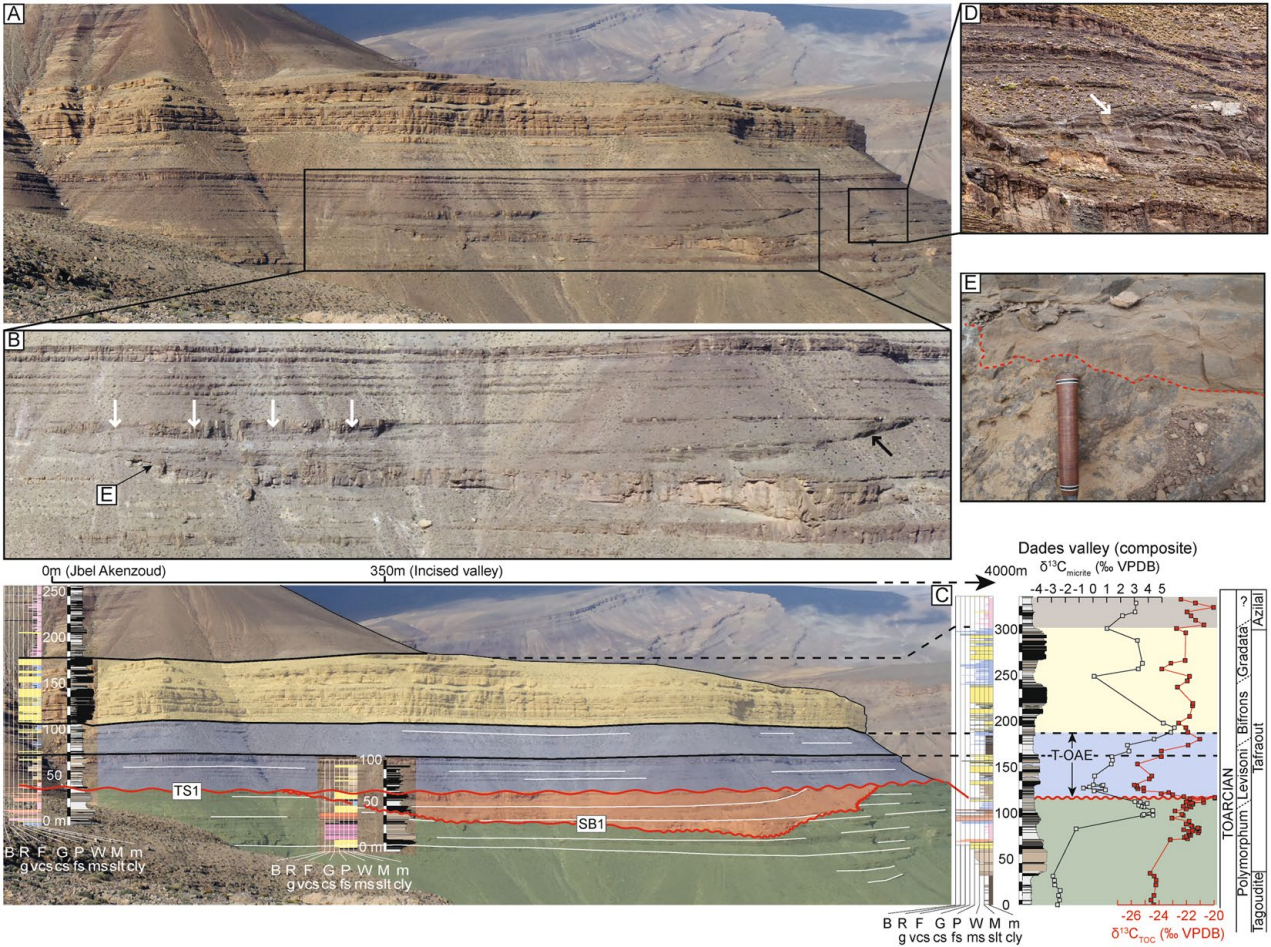
(**A**) General aspect of the outcrop from Jebel Akenzoud (Dades Valley, Central High Atlas Basin, Morocco). The two rectangles indicate zoom in on selected portions of the outcrop. (**B**) Close-up view of the left-hand side of the panel. Lateral accretion within the ooidal shoal in the uppermost part of the infilling unit can be seen (white arrows). The black arrow points at the right edge of an inferred tidal channel within the uppermost infilling unit. (**C**) Stratigraphic correlation of sections TS1 and SB1 (this study) with Dades Valley composite^13^. From the bottom to the top, the green unit = lower host unit; orange unit = median infilling unit; blue unit = upper sealing unit, stratigraphically correlative to the T-OAE; yellow unit = rest of the sealing unit. Note that the 50 m incision affects the uppermost part of the Polymorphum Zone and is directly overlain by sedimentary rocks belonging to the T-OAE stratigraphic interval. (**D**) Close up view on the right-hand side shoulder of the infilling unit. The presence of a wedge-shaped sedimentary body (white arrow) hints at multiple sub-episodes of erosion and deposition during the infill of this unit. (**E**) Close up on the erosion surface affecting the lower host unit (SB1).

Panoramic observation of the exposure at Jebel Akenzoud reveals the presence of a wide valley-shaped incision, ~50 m-deep within the lower part of the early to middle Toarcian Tafraout Formation (Figs 2 and S1). The succession can thus be divided into three main units delimited by two discontinuities: sequence boundary 1 (SB1) and transgressive surface 1 (TS1). From the bottom to the top, these units are: (i) the host unit; (ii) the infilling unit; and (iii) the sealing unit.

The host unit (green in Fig. 2C) is laterally continuous and starts with interbeds of claystone and fine-grained sandstone interpreted as turbidites, which belong to the Tagoudite Formation^13,49,50^. Progressively, the Tagoudite Formation is replaced by the mixed carbonate/siliciclastic Tafraout Formation^51^. Within this formation, limestone beds are mainly ooid-rich grainstone showing common flaser bedding, herringbone cross stratification, and/or oscillation ripple. Three different types of boundstone are included within the host unit and correspond either to coral or lithiotid bafflestones, or microbial bindstones. The siliciclastic phase is mostly dominated by soft recessive intervals of claystone and polymictic conglomerates. The latter ones contain sub-angular bioclasts and extraclasts of metamorphic rocks and rounded to sub-rounded intraclasts of vuggy mudstone, ooid-rich grainstone, and glauconite. Observed clasts are never larger than 5 cm and laterally evolved down-dip toward arenites and lutites within a distance of 4 km. Common sedimentary features observed within this clastic-dominated stratigraphic interval are current ripple and hummocky cross stratification (HCS).

The infilling unit (orange in Fig. 2C) is laterally discontinuous and was deposited in an asymmetric trough with a maximum width and height of about 700 m and 50 m, respectively. The trough belongs to the Tafraout Formation and is filled in its left part with several-meters-thick amalgamated ooid-rich grainstone beds separated from SB1 by a 5 m thick soft recessive claystone interval. The ooid-rich grainstone show common flaser bedding, herringbone cross stratification, and/or oscillation ripple. In the uppermost 15 m on the left-hand side of the infilling unit, lateral accretion can be observed within the ooid-rich deposits (Fig. 2B) interpreted as tidal channel infill^52^. The difficulty to access this part of the cliff has so far prevented further in-depth investigation of the infilling unit.

**Figure 3:**
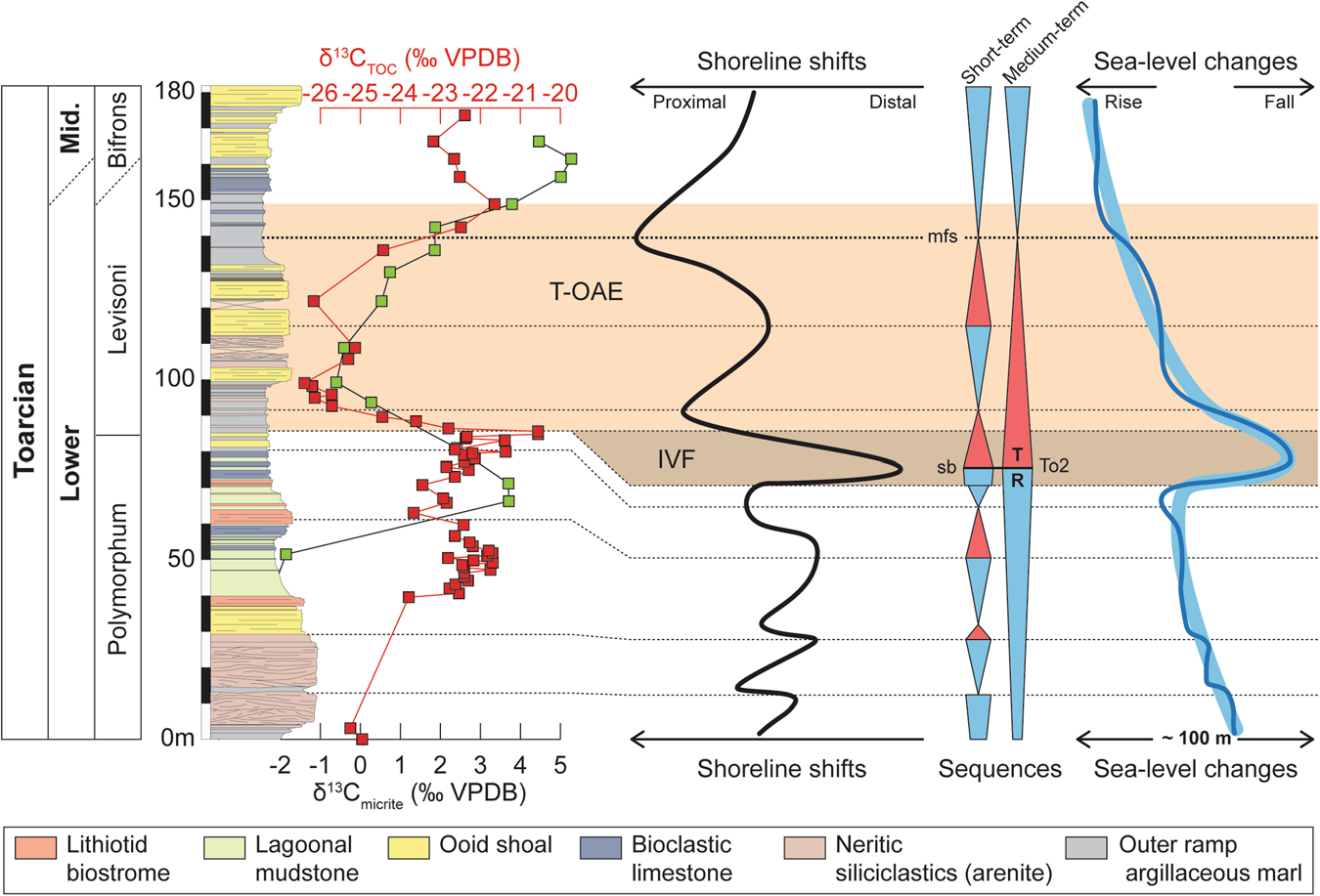
Summary of shoreline shifts and inferred sea-level changes as deduced from the Dades Valley record. The time interval encompassed by the infilling unit is lacking on the Dades Valley composite section (hiatus surface) as this later was logged in Jebel Toksine, ca. 4 km further downdip from the Jebel Akenzoud^13^.

The sealing unit caps the underlying units and can be divided into two members. The bottom part of the lower member (blue in Fig. 2C) is dominated by tempestites, interpreted as such based on the common occurrence of fine-grained sandstone to siltstone beds, showing HCS features^13^. The lower member becomes upward progressively dominated by ooid-rich grainstone interpreted as tidalites based on the common occurrence of flaser bedding, herringbone cross stratification and oscillation ripples. The upper part of the lower member is represented by a soft recessive claystone interval, indicative of an important deepening event (Fig. 3). The upper member of the sealing unit (yellow in Fig. 2C) consists of two stacked coarsening-upward units (Fig. 2), starting at the base with 30m-thick deeper-water marine claystone intervals with numerous interbeds of bioclastic wackestone to packstone. The faunal content of the limestone bed consists of bivalve, echinoderm (including *Pentacrinus*), brachiopod, and coral debris. Claystone intervals contain foraminifera and ostracods^50^. Toward the top of each coarsening-upward units, claystone intervals are gradually replaced by shallow marine ooid-rich grainstone showing flaser bedding, herringbone cross stratification and oscillation ripples. This upper member corresponds to the upper part of the Tafraout Formation of middle Toarcian age^13,50^. No angular unconformity between the strata of the host and the sealing units is observed.

#### Choronostratigraphic framework

According to the chronostratigraphic framework for the Toarcian of the Dades Valley^13,53^, the wide valley-shaped incision was created and capped during the earliest Toarcian, before the onset of the T-OAE (more precisely during the late Polymorphum Zone). This age attribution is confirmed by the occurrence of the brachiopods *Soaresirhynchia bouchardi* and *Pseudogibbirhynchia jurensis* in the lower part of the sealing unit in the Ouguerd Zegzaoune section (at the height 150 m in the log figuring in ref.^13^). These two brachiopods are indeed only found in association in the western Tethyan realm within the Levisoni Zone^54–56^, confirming that the negative carbon isotope excursion recorded within the sealing unit^13^ corresponds to the one associated with the T-OAE^2^.

#### Sequence stratigraphic interpretation

**Figure 4:**
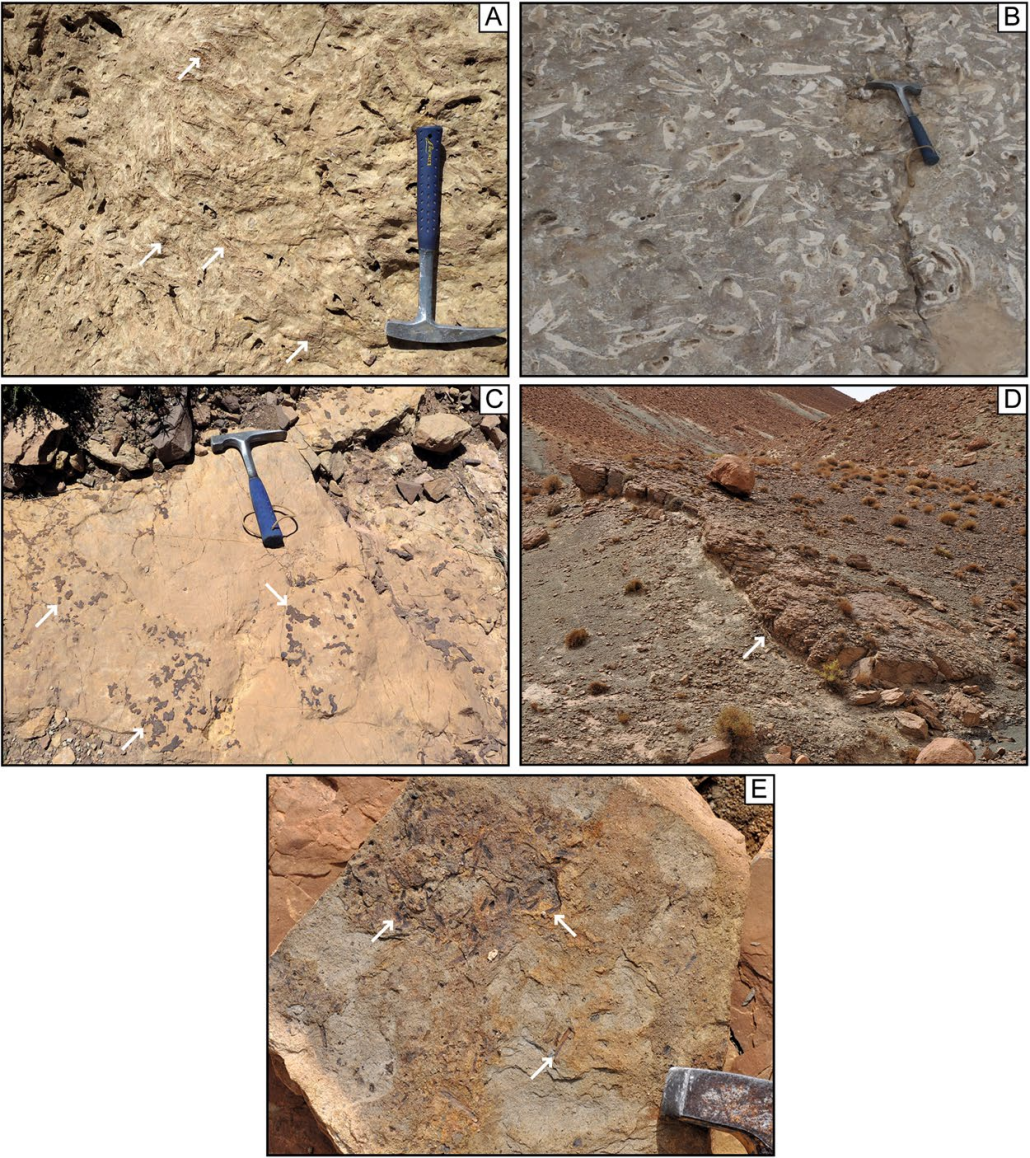
(**A**) Dissolved lithiotid shells filled by vadose silts (white arrows) and embedded within a dolomitized matrix, below SB1 in proximal area, 350 m northwest of the infilling unit (Jebel Akenzoud, Dades Valley). (**B**) See non-dissolved lithiotid shells for comparison. (**C**) Dolomitic bed associated with SB1 in Taria n’Dades (most proximal section visited during this study). Note the iron oxides veneer (white arrows) on top of the dolomitic bed corresponding to TS1. (**D**) 40-cm-thick channelized packstone bed (max. 1 m thick; white arrow) composed of shallow-water components interbedded within deep-neritic marls (Amellago section). This feature is interpreted as a local shelf margin wedge associated with SB1. (**E**) Numerous plant debris (white arrows) are present within this bed.

The host unit, the infilling unit, and the sealing unit are separated by two key surfaces that, based on their characteristics, are interpreted as a sequence boundary (SB1) and a transgressive surface (TS1), respectively^57^ (Figs 2 and S1). SB1 separates the host and the sealing units and is characterized by the presence of karstification materialized by the dissolution of lithiotid shells in the beds upon which it rests (Fig. 4A, see also Fig. 4B for undissolved lithiotid shell comparison). No cavities are associated with the karstified surface. However, the underlying carbonate beds show evidence of dissolution, recrystallization and infill of vadose silts within a 10-meter interval down section (Fig. 4C), a feature specific to this stratigraphic interval in the Dades Valley. Laterally, SB1 corresponds to a 50-meter deep incision into the host unit (Figs 2 and 4B). The stratigraphic distribution of the karstified surface, the 10-meter thick dolomitized horizon, and the incision strongly suggests a subaerial exposure of the Tafraout shallow marine carbonate during the latest Polymorphum Zone forced regression. SB1 has an asymmetric trough-shaped geometry. Numerous on-lapping terminations are observed at the contact between SB1 and the sedimentary rocks, which constitutes the infilling unit (Figs 2 and S1).

TS1 corresponds to a major change from regressive to transgressive trend. In the field, this is evident as a stack of deep-neritic claystone facies on top of a subaerial unconformity (SB1), which is a clear signature for a landward migration of the shoreline^57^ (Fig. 3). A chaotic iron-manganese-oxide crust characterizes TS1 in more proximal settings (Fig. 4C). In distal areas, this surface is marked by *Glossifungites* ichnofacies. The dominant ichnogenera are firmground *Skolithos*, *Arenicolites* and *Diplocraterion*. Noteworthy, TS1 is horizontally flat and seals the host and infilling units. On both sides of the infilling unit, TS1 and SB1 are coincident.

### Amellago

#### Stratigraphy

In the distal neritic setting of Amellago, a conspicuous 1-m thick channelized packstone bed occurs within the Tagoudite Formation, 45 m above the uppermost limestone bed of the Ouchbis Formation, which is dominated by carbonate mudstone-marl alternations^18^ (Fig. 4D). This peculiar bed is characterized by numerous shallow-water components and large plant debris (Figs 4D and 4E). These features contrast with the surrounding clay-dominated sedimentary rocks of the Tagoudite Formation, best assigned to deep-neritic depositional setting^48^. This conspicuous channelized packstone bed is interpreted as a local shelf margin wedge, deposited during the late Polymorphum Zone.

#### Chronostratigraphic framework

In Amellago, the age assignment of the section is based on nannofossil and ammonite biostratigraphy, complemented by carbon isotope chemostratigraphy^18,48^.

The three uppermost beds of the Ouchbis Formation are characterized by the common occurrence of the ammonite *Dactylioceras* sp. This biostratigraphic event marks the beginning of the Toarcian (Elmi, 2006) and occurs right below the abrupt lithological change between the Ouchbis and the Tagoudite Formations^18^. Approximately 30 meters above the Toarcian–Pliensbachian boundary, the first occurrence of the nannofossil *Carinolithus superbus* is recorded. This event is significant because it is indicative of the Polymorphum Zone^58^. This interpretation is consistent with the *Harpoceras serpentinum* ammonite specimen, found 60 meters above the Pliensbachian–Toarcian boundary and supported by the record of the T-OAE carbon isotope excursion few meters above the *H. serpentinum* finding^18,48^.

## Jameson Land, East Greenland

The Lower Jurassic (Pliensbachian–Toarcian) Neill Klinter Group of the Jameson Land Basin (Fig. 1) has been extensively studied for its paleontological content, sedimentological facies and sequence stratigraphy^59–64^. These studies have highlighted the evidence for several regional episodes of sea-level falls within the Neill Klinter Group, as inferred from the recurrent incised valley/estuary complexes within the Ostreaelv Formation. Nevertheless, a precise timing for the onset and termination of these sea-level fluctuations has never been achieved and the sequence stratigraphic framework was not accurately constrained due to the scarcity of marine fauna. As such, an accurate correlation with other basins’ sea-level evolution is presently not feasible, precluding any interpretation on the local vs. global drivers behind relative sea-level changes. This is nonetheless important since ample (up to 50 m) sea-level fluctuations have been inferred^59,62,63^.

### Stratigraphy

**Figure 5:**
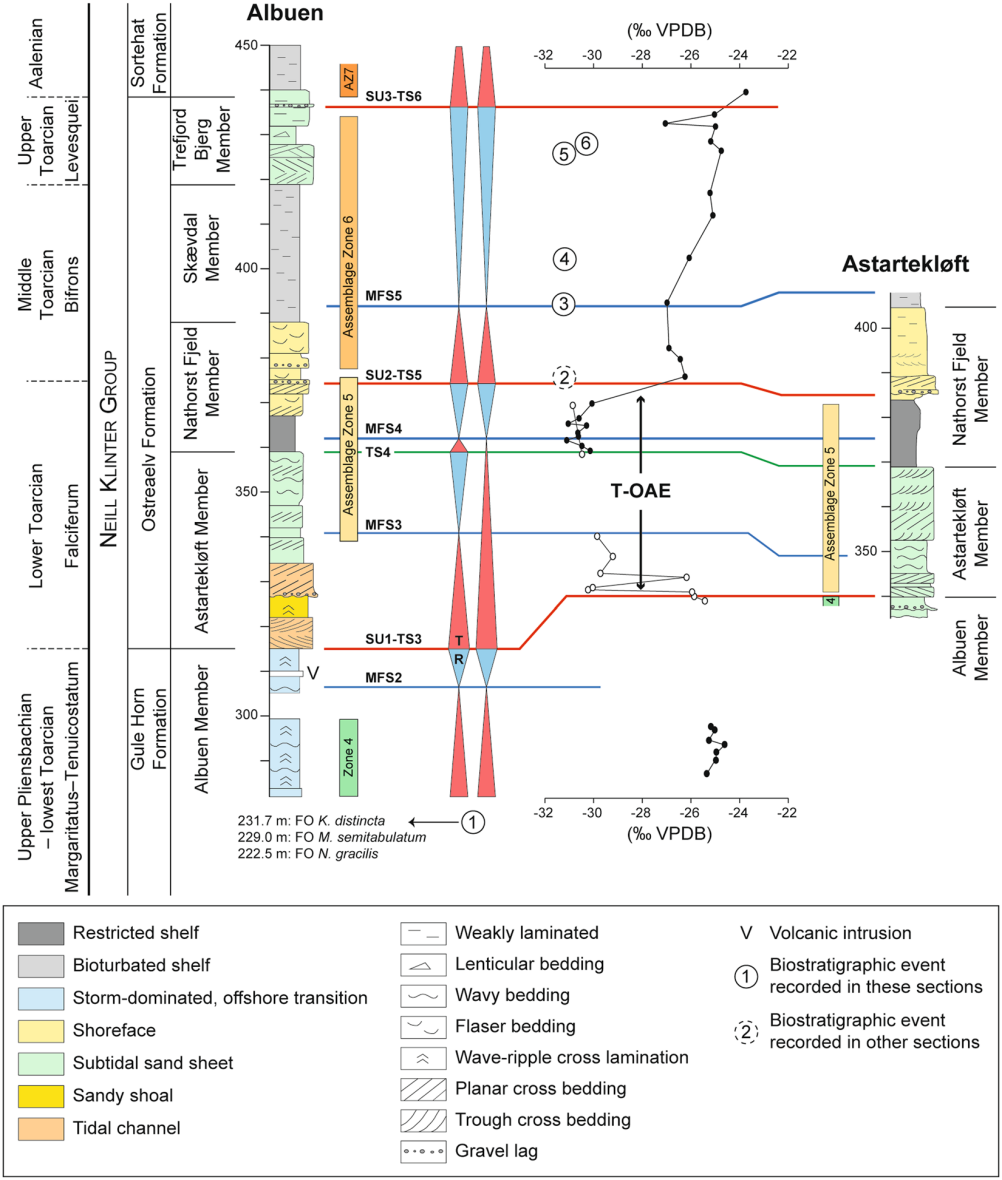
Lithostratigraphy and biostratigraphy of the Albuen and Astartekløft sections (modified after ref.^61^). The assemblage zones refer to the palynomorph assemblage zones of Koppelhus and Dam^61^: Assemblage zone 4: Upper Pliensbachian, Assemblage zone 5: Lower Toarcian *s.l*., Assemblage zone 6: Upper Toarcian *s.l*., Assemblage zone 7: Aalenian. Biostratigraphic events: (1) First occurrences (FO) of *Nannoceratopsis gracilis, Mancodinium semitabulatum* and *Kekryphalospora distincta*; (2) FO of *Parapassolotheuthis polita*; (3) FO of *Parvocysta* sp.; (4) FO of *Parvocysta eumekes*; (5) FO of *Parvocysta elongata*; and (6) FO of *Callialasporites* spp. The lithostratigraphic units and sequence stratigraphic interpretation are following the interpretations of Ahokas *et al*. (ref.^63^). For the carbon isotope curve, the black dots represent analyses from the Albuen section, and the white dots analyses from the Astartekløft section.

The Neill Klinter Group is clastic-dominated and is subdivided into four formations^62,64^. In stratigraphic order those are the Ræveløft, Gule Horn, Ostreaelv, and Sortehat Formations. In this study, the Ræveløft Formation is absent and therefore will not be further discussed (Fig. 5). The Gule Horn Formation includes two members, the Albuen and Elis Bjerg Members. The Albuen Member forms the uppermost part of the Gule Horn Formation located at the southern part of the Jameson Land. This member is characterized by brackish-marine embayment deposits replaced towards north by the sandy heterolithic Elis Bjerg Member^62,64^. In the southern part of Jameson Land, the Ostreaelv Formation includes the Astartekløft, Nathorst, Skævdal, and Trefjord Members and is composed of interstratified heterolithic marginal-marine sandstones and mudstones best assigned to paralic environments each deposited at or near sea-level^62,64^. The uppermost part of the Neill Klinter Group is the mudstone-dominated Sortehat Formation, which has been interpreted as deep-neritic claystone^62,64^.

### Chronostratigraphic framework

Using samples stored at the Geological Survey of Denmark and Greenland (GEUS), that were originally collected for a palynological study by Koppelhus and Dam (ref.^61^), we have undertaken carbon isotope analyses on bulk organic matter on two sections, namely the Albuen and Astartekløft sections. The use of these two sections offers a complete coverage of the uppermost Pliensbachian–lowermost Aalenian (Fig. 5). Departing from the ca. –25‰ background values, organic matter carbon isotope results clearly show that a large negative carbon isotope excursion starts in the lowermost Astartekløft Member, reaching –31‰ in its uppermost part, ending in the middle part of the Nathorst Fjeld Member.

The Gule Horn Formation is assigned to the Pliensbachian, with the upper part of the Elis Bjerg Member and the Albuen Member assigned to the upper Pliensbachian–lowermost Toarcian (Margaritatus–Tenuicostatum, Polymorphum equivalent, Zones) *Luehndea spinosa* Zone, based on the co-occurrence of the dinoflagellate cysts *Nannoceratopsis gracilis* and *Mancodinium semitabulatum*^61^. Koppelhus and Dam^61^ interpreted the presence of *Luehndea spinosa* (known range encompassing the Margaritatus–Tenuicostatum, Polymorphum equivalent, Zones^65^) in the basal part of the Nathorst Fjeld Member as indicating the lower Toarcian Tenuicostatum, Polymorphum equivalent, Zone^61^. However, they also documented the presence of *L. spinosa* in the middle part of the Nathorst Fjeld Member, and in the Trefjord Bjerg Member (Fig. 4B in ref.^61^). In addition, *Valvaeodinium armatum*, with a known last occurrence in the Spinatum Zone^65^ was documented in the upper part of the Nathorst Fjeld Member^61^. The belemnite *Parapassolotheuthis polita* suggests that part of the Nathorst Fjeld Member is assigned to the uppermost Falciferum, Levisoni equivalent–lowermost Bifrons Zones^61^, i.e. the transition between the lower and middle Toarcian. The fact that *V. armatum* and *L. spinosa* occur in reverse order within the Nathorst Fjeld Member suggests that they are both reworked. Koppelhus and Dam^61^ (Figs 4a and 10 in ref.^61^) did note the occurrence of reworked palynomorphs taxa of Carboniferous to Triassic age within the Nathorst Fjeld, the Skævdal and the middle Trefjord Bjerg Members (Fig. 6A in ref.^61^). The first occurrences of *Parvocysta* sp. and *P. eumekes* in the lower and middle Skævdal Member, and *Parvocysta elongata* in the lower Trefjord Bjerg Member, suggests that these units are not older than the Bifrons and Pseudoradiosa Zones, respectively^61,65^. An early late to late Toarcian age for the lower Trefjord Bjerg Member is further confirmed by the first occurrence of *Callialasporites* spp. at this level^66^. Altogether, this constrains the age of the negative carbon isotope excursion recorded in the Astartekløft Member to the early Toarcian. Given its large amplitude (−6‰ shift), it is equated to the T-OAE negative carbon isotope excursion^2,15,18^.

### Sequence stratigraphic interpretation

Following the sequence stratigraphic scheme by Ahokas *et al*.^62,63^ (Fig. 5), this negative carbon isotope excursion is bounded between the SU1 and SU2 surfaces and encompasses sedimentary rocks of the Astartekløft and the lower part of the Nathorst Fjeld Members. The Astartekløft Member was formed during a large transgressive interval and resulted in infilling of the valley-like topography created during a forced regression that must have eroded at least 28 m of the Gule Horn Formation, and thus occurred between the deposition of the Albuen and Astartekløft Members^62^.

This highlights that in East Greenland, in a similar manner as in Morocco, the T-OAE is preceded by a major sea-level drop of at least 28 m as deduced from the sedimentology and sequence stratigraphy. Moreover, the T-OAE took place mostly during the subsequent transgression associated with a major sea-level rise. However, the lowstand system tract is not recorded in the siliciclastic setting of onshore East Greenland, which contradicts with the results obtained from Morocco. Finally, one can notice that in East Greenland, in a similar manner as in Morocco, the stratigraphic interval encompassing the T-OAE transgression is interrupted by a relatively short-lived episode of normal regression^62,63^ (Fig. 5). This underlines the likelihood that the sedimentation rate outpaced the rate of sea-level rise during the T-OAE.

## A Eustatic Sea-Level Swing During the Earliest Toarcian?

The Moroccan and East Greenland examples highlight that the early Toarcian long-term transgression was briefly interrupted, during the late Polymorphum, Tenuicostatum equivalent, Zone, by a normal regression followed by a brief and large amplitude base-level fall. Pieces of evidence for normal regression in the upper Polymorphum Zone are numerous in Tethyan localities, where it has been firmly reported in France^40^, Italy^41^, Poland^42^, England^29^ and Portugal^43^. In each case, the regression shortly precedes the onset of the T-OAE recorded in the upper Polymorphum Zone, with some evidence for a hiatus or condensation in the uppermost part of the Polymorphum Zone. We emphasize here that this regression should not be confused with the one characterizing the uppermost Pliensbachian^38,43^, a confusion that could arise from the fact that the Polymorphum Zone is commonly highly condensed, if not lacking, in numerous sections^67,68^. This condensation also often hampers a proper characterization of sea-level fluctuations in these localities.

Examples of normal regression in the upper Polymorphum Zone are well documented in the literature, but there is little evidence for forced regression. Hence, outside of Morocco and East Greenland, only circumstantial evidence for a late Polymorphum Zone forced regression were proposed for the Toarcian of Portugal^43^, whereas in the North Sea, several poorly-dated deep incised valleys within the Toarcian Cook Sandstone^69^ await further high-resolution bio- and chemostratigraphic studies in order to be firmly correlated to the ones described here.

### Timescale of the forced regression

The duration of the Polymorphum Zone has been estimated to about 1 Myr^70–72^. It is possible to infer the duration for the forced regression based on the correlation of discontinuities between the Central High Atlas and the Lusitanian Basins (Portugal). Indeed, the discontinuities SB1/TS1 (Morocco) and D2^43^ (Portugal) are both recorded in platform settings within the topmost Polymorphum Zone, directly below the T-OAE carbon isotope excursion. Based on SB1/TS1 and D2 stratigraphic similarities, we suggest that both discontinuities result from the latest Polymorphum sea-level fall. In the Lusitanian Basin, D2 corresponds to a subaqueous erosion surface triggered by fairweather and/or storm waves action and is only recorded in platform settings^43^. In deeper settings (below the storm weather wave action), such as the one recorded at the Peniche section (Toarcian GSSP), D2 is absent, but corresponds to a ca. 5 m-thick stratigraphic interval based on chemostratigraphic correlation^43^. Based on two divergent cyclostratigraphic studies carried on the Peniche section, the duration of the sea-level fall (D2) is thus estimated to be either 105 kyr or 250 kyr (depending on the cyclostratigraphic scheme applied from ref.^44^ or ref.^73^, respectively). It has been suggested that the early Toarcian in Peniche is incomplete^44^. The time missing in this section is however unlikely to be associated with the forced regression since this system tract, together with the lowstand system tract, is usually characterized by the highest sediment delivery and accumulation in basinal setting^74^. We suggest that in Peniche, hiatuses are most likely associated with the condensed intervals documented at the Pliensbacian–Toarcian boundary and within the onset of the T-OAE carbon isotope excursion^43,44^. In summary our best estimate for the duration of the latest Polymorphum sea-level fall is at least 100 kyr, and possibly longer given the likelihood of the presence of hiatuses or extreme condensation in the Peniche section^43,44,72^.

### Triggering mechanisms to the latest Polymorphum forced regression

Evidence for a short-term (≪500 kyr) forced regression has been recognized in Morocco and East Greenland. According to Sames *et al*.^75^, mechanisms able to explain such sea-level changes are restricted to: (1) dynamic topography, (2) aquifer-eustasy, and (3) glacio-eustasy. This is because the timescales at which other mechanisms are operating are either too long (e.g. changes in sea-floor spreading rates) or the orders of magnitude in eustatic sea-level change is out of range of the one recorded in Morocco and East Greenland (e.g. thermal expansion of the oceans, Table 1). Possible mechanisms that may or may not explain this latest Polymorphum forced regression are discussed below.

**Table 1:** Overview of potential mechanisms that may or may not have influenced sea-level change during the Polymorphum Zone.

Mechanisms/Event	Operative timescales (kyr)	Orders of magnitude in eustatic sea level (m)	Potential extent	Polymorphum forced regression relevance
Polymorphum forced regression	**<500**	**>50**	**Global**	High
Thermal expansion (thermo-steric effect)	**1 to 10**	~5 to 10	**Global**	Average
Continental glaciations/deglaciations	**<10 to 100**	**~50 to 250**	**Global**	High
Continental groundwater storage and release	**<10**	**~10 to 50**	**Global**	High
Dynamic topography	**0.1 to 100**	**Up to 100**	Regional	Average
Other tectonic deformations	≥1000	**~10 to 1000**	Regional/global	Low

The characteristics in common with the Polymorphum regression event are represented in bold (modified from Sames *et al*.^75^).

### Dynamic topography

As both eustasy and dynamic topography (or any other solid-earth processes that affect regional topography) can operate on a 10^2^ kyr time scales, it is necessary to ensure that a sea-level fluctuation is coeval over several basins in order to infer their eustatic origin^76^. This is due to the fact that solid-earth processes produce sequences that correlate only over a few hundreds of kilometers^77^. A major difficulty in inferring coeval sea-level variations in the deep past comes from the chronostratigraphic resolution that is often of too low for unambiguous correlation^76^, especially in shallow marine or continental deposits where reliable biostratigraphic markers are scarce. Nevertheless, the large negative carbon isotope shift characterizing the onset of the T-OAE is a prominent chemostratigraphic marker that allows correlation between lower Toarcian marine and continental deposits^2,31,43^. Hence, within the current limits of bio-chemostratigraphic resolution, there is a similar pattern of sea-level change reported from the upper Polymorphum Zone in Morocco, Portugal and East Greenland, over paleogeographically reconstructed distances greater than 3000 km.

One might still argue that similar tectonic forcing occurred at the same time in Morocco and East Greenland as a result of coincidence. Tectonic forcing acts on the total volume of the basins under consideration by either creating topographic highs or lows. This would then imply an angular unconformity between the host unit and the infill unit. In both Morocco and East Greenland there is no stratigraphic evidence that this incision is linked to tectonic activity such as tilting, faulting or folding of the part of the section located below the documented incisions^62^ (see Figs 2 and S1). We therefore rule out tectonics as the main driver of relative sea-level fluctuation, reflected by the common stratigraphic patterns observed in Morocco and East Greenland.

### Aquifer-eustasy

Currently, there are two continental reservoirs able to store effectively large volume of water removed from the ocean: (1) aquifers and (2) continental ice sheets^75^. It has been suggested that during periods of the Earth history when paleoclimatic conditions were prohibiting the formation of large continental ice sheets (warm greenhouse and hot-house intervals, e.g. Cenomanian–Turonian), short-term sea-level fluctuations would primarily be controlled by the amount of water displaced from the ocean and stored in continental aquifers^78^.

The present-day reservoir capacity of continental groundwater is estimated at 25 × 10^6^ km^3,78^. Filling or emptying entirely this reservoir would allow sea-level changes of 50 meters^78^. However, the size of the reservoir is fluctuating through time as a consequence of long-term sea-level changes (≫1 Myr), which have a major influence on the size of the ocean basin volumes. For instance, during the Cenomanian sea-level highstand, the intense activity of mid ocean ridges generated an average sea level 200 meters higher than present day^79^. This implies a larger volume of flooded landmasses and therefore a larger size for aquifers. It has been estimated that during the Cenomanian, the size of the groundwater reservoir could have been twice that of today allowing sea-level variations of about 80 meters instead of 50 meters^78^ (present day). During the early Toarcian, the mean sea level is estimated to be similar to the present day one^80^. Consequently, there is no reason to think that aquifer-eustasy would trigger more than 50 meters of sea-level fluctuations^81^. In Morocco, the Jebel Akenzoud transect shows a ca. 700 m wide structure that corresponds to an incised-valley fill, created by at least 50 meters of sea-level drop. This is a minimum estimate since sediment compaction has not been considered, and that there is no certainty that the 50 m-deep incision observed here represent the maximum base-level amplitude. It is therefore questionable to consider aquifer-eustasy as the most likely mechanism to explain the field observations.

Moreover, in the aquifer-eustasy model the eustatic sea-level changes are imposed by the balance between the amount of inland precipitation (continental input) and the efficiency of drainage systems (rivers and aquifers, continental output) to redistribute this water into the ocean^82^. Consequently, sea-level falls happen when the amount of precipitation exceed the fluvial runoff during time of enhanced hydrological cycle associated with warm greenhouse and hot-house intervals^82,83^. Several lines of evidence support the scenario of cool and dry conditions in the European sections and Siberia during the Polymorphum Zone, which is incompatible with the aquifer-eustasy sea-level fall (See section below).

### Glacio-eustasy

Due to the absence of undisputable evidence for polar ice sheet during the Mesozoic, the existence of glaciation under this overall greenhouse climate remains vigorously debated^4,82,84,85^. Nonetheless, paleotemperature reconstructions based on oxygen isotopes in marine invertebrates have shown that cool climatic conditions prevailed at several times during the Jurassic^3,4,11,86,87^, notably just prior to the T-OAE during the late Polymorphum Zone^12^. This is supplemented by terrestrial plant fossil data that indicate relatively low atmospheric pCO_2_ levels (around 500 ppm) during the earliest Toarcian, before the T-OAE^31^. Paleoclimate modeling indicates that these pCO_2_ values are compatible with the transient development of Mesozoic ice sheet when coupled, for instance, with episodes of minimal polar summer insolation^88,89^. Indirect sedimentary evidence for cool marine temperatures such as glendonites has been reported in Siberia below the T-OAE stratigraphic interval^24^. In summary, given the growing body of evidence indicating cooler climate conditions prior to the T-OAE, and despite the absence of direct sedimentological record for glaciation, but also given the absence of an alternative mechanism, we therefore posit that the late Polymorphum sea-level swing is most likely linked to the transient development of polar continental ice. It remains as yet uncertain which mechanism could have caused this transient cooling, but enhanced burial of organic matter on a global scale might be a possible candidate since the upper Polymorphum Zone is characterized by a positive carbon isotope shift^2,3^. Alternatively, massive and sustained emission of volcanic aerosols during an eruptive pulse of the Karoo-Ferrar large igneous province might also be invoked^90,91^, although the absence of Hg enrichment in the upper Polymorphum Zone^28^ is not in favor of this second hypothesis.

## Consequences of a Major Glacio-Eustatic Sea-Level Fall Shortly Before the T-OAE

The potential existence of a late Polymorphum “cold snap” has further implications for the understanding of the T-OAE and its causes. Indeed, in order to explain concomitant atmospheric and oceanic changes in carbon cycling associated with negative carbon isotope excursion^1^ a massive and relatively sudden input of gas hydrate into the ocean-atmosphere has been invoked. However, to date there is little understanding on the modality of where gas hydrate might have been stored in or released from during the Toarcian. Recent studies show no consensus concerning the size of the current day gas hydrate reservoirs. Estimations range from 1600 to 2000 gigatons of carbon (GtC) in the ocean and 400 GtC in the Arctic permafrost^92^. Due to the pressure and temperature dependency of the gas hydrate stability zone, the permafrost reservoir is highly sensitive to global warming as attested by recent studies^78^. On the contrary, the ocean reservoir for gas hydrate and more precisely gas hydrate located in deep-sea setting are supposed to be less sensitive to climate change^93^. This is due to the fact that the deep-sea reservoir is located thousands of meters below the oceanic thermal mixing zone. Therefore, a geologically unreasonable warming temperature (~20 °C) at water depth of about 1500 m or more is required to dissociate gas hydrate belonging to this reservoir^93^. We speculate that thawing of permafrost, due to the initial warming from massive release of volcanic CO_2_ from the Karoo-Ferrar volcanic province, acted as a positive feedback during the T-OAE global warming. The highly ^13^C-depleted signature (−40 > δ^13^C > −100‰) of methane locked in permafrost could explain, at least partly, the marine and atmospheric negative carbon isotope shift characteristic of the onset of the T-OAE. The cold conditions reported prior to the T-OAE^12^ might explain how these hydrates accumulated in a first instance under an overall greenhouse climate^93,94^. The permafrost hypothesis and how it might have influenced the early Toarcian environmental disturbances has already been evoked previously^45,95^ but relied on less substantial arguments for cold climate conditions. In this study, we bring an exceptional sedimentological record of an ample and rapid sea-level fall, constrained by a robust chronostratigraphical framework, that can only be confidently associated with glacio-eustasy according to our current understanding of global sea-level fluctuation mechanisms. Hence, this dataset highlights how the T-OAE hyperthermal might have been rooted in past cold-house climate. As such we provide a outstanding constraint on glacio-eustasy during the Jurassic “greenhouse”.

## Methods

### Field work approach and petrography

Two stratigraphic sections were measured in the Central High Atlas (Fig. 1). These sections S1 and S2 are located on the north-western flank of the Dades Valley (GPS coordinates S1: N31°37′17.7″; W5°53′33.8″; GPS coordinates S2: N31°37′9.92″; W5°53′24.07″). The section S1 complete the lower part of the Jebel Akenzoud section described in ref.^11^. A total of about 200 m of Lower Jurassic sedimentary rocks were logged and described bed by bed. The focus was on lateral as well as stratigraphic facies changes, sedimentary features and textures, biota, trace fossils and diagenetic features. Facies table and color-coding for outcrop sections in Fig. 2 are given in supplementary material (Fig. S2).

### Bulk organic matter Carbon isotope analyses

Carbon isotope analyzes of the total organic carbon (δ^13^C_TOC_) were performed at Erlangen University on 40 de-carbonated samples from the Albuen and Astartekløft sections (East Greenland). Powdered samples were treated two times with 6 M HCl for 12 h to remove any carbonate phases and rinsed subsequently with deionized H_2_O until neutrality was reached at Bochum University. Carbon isotope analyses of organic carbon were performed with a Flash EA 2000 elemental analyser connected online to ThermoFinnigan Delta V Plus mass spectrometer. All carbon isotope values are reported in the conventional δ-notation in permil relative to V-PDB (Vienna-PDB). Accuracy and reproducibility of the analyses was checked by replicate analyses of laboratory standards calibrated to international standards USGS 40 and 41. Reproducibility was ±0.06‰ (1σ).

## Supplementary information

A major sea-level drop briefly precedes the Toarcian oceanic anoxic event: implication for Early Jurassic climate and carbon cycle
