# Supplementary material for: A major sea-level drop briefly precedes the Toarcian oceanic anoxic event: implication for Early Jurassic climate and carbon cycle
Source: Sci Rep. 2019 Aug 29;9:12518. doi: 10.1038/s41598-019-48956-x (PMC6715628; doi:10.1038/s41598-019-48956-x)
Supplement: Supplementary file 1 — A major sea-level drop briefly precedes the Toarcian oceanic anoxic event: implication for Early Jurassic climate and carbon cycle [file 41598_2019_48956_MOESM1_ESM.pdf]

A major sea-level drop briefly precedes the Toarcian oceanic anoxic event: implication for Early Jurassic climate and carbon cycle

François-Nicolas Krencker[1]*, Sofie Lindström[2], Stéphane Bodin[1]

[1] Department of Geoscience, Aarhus University, Høegh-Guldbergs Gade 2, 8000 Aarhus C, Denmark
[2] GEUS–Geological Survey of Denmark and Greenland, Øster Voldgade 10, DK-1350 Copenhagen K, Denmark

* Corresponding author: fkrencker@geo.au.dk

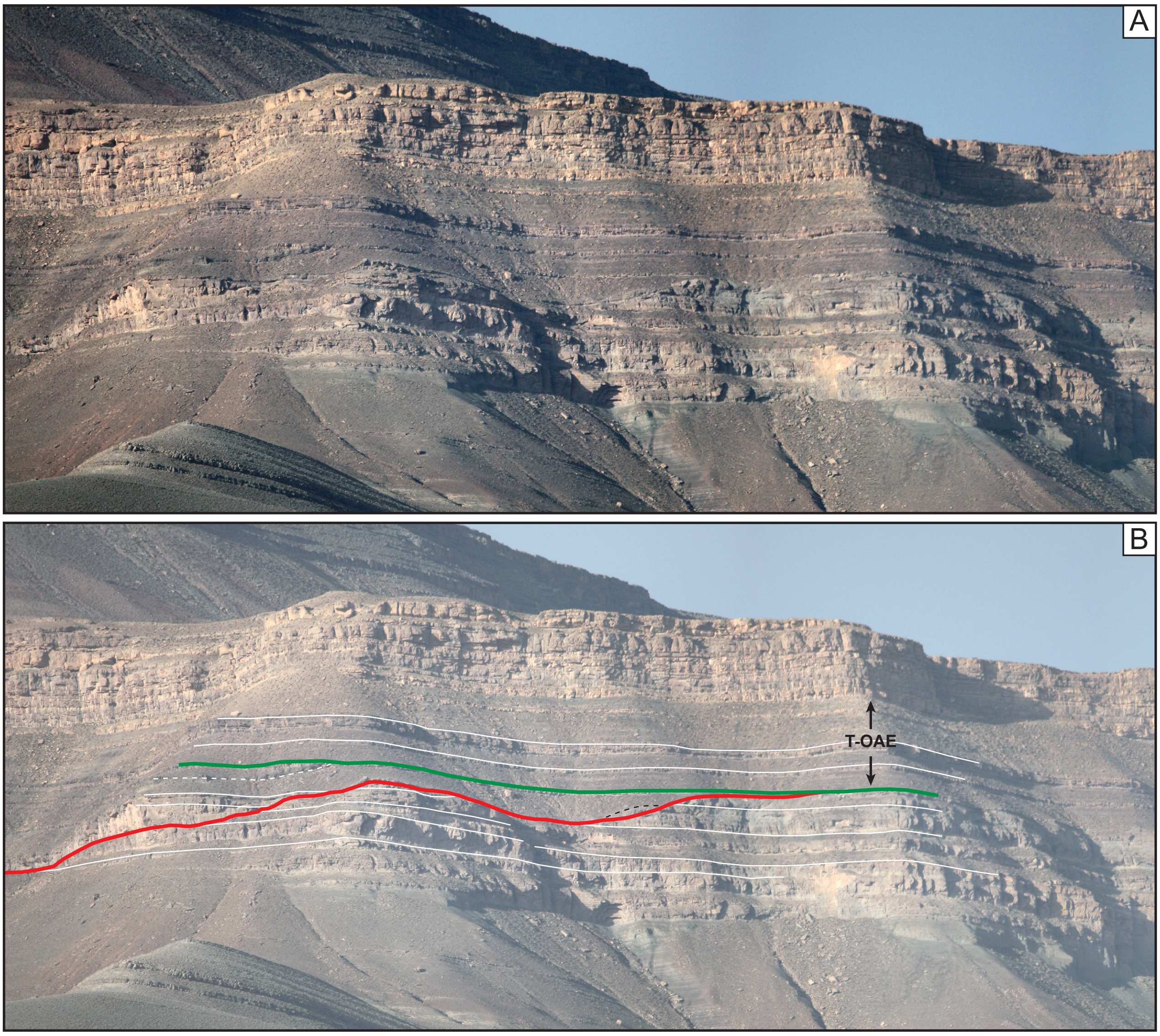

Fig. S1: General aspect of the wide valley-shaped incision (~ 50 m-deep) from Jebel Akenzoud (Dades Valley, Central High Atlas Basin, Morocco) without (A) and with (B) line drawing.

Color code	Facies/Key biota	Texture	Allochems	Sedimentary structures	Depositional environment
1a	Laminites and mudflat facies	W, M-W	Microbial mat 2, peloids 2, plant debris 2, lithoclasts (glauconite) 2	tepee structures, vugs	Tidal mudflat
1b	High-energy facies	G, P	Ooids 4, bioclasts 1-2, lithoclasts (quartz, glauconite) 1-2, peloids 1-2	tidalites, flaser bedding, wave ripples, trough cross bedding	Tidal sandflat
2a	Bivalve (Lithiotids) biostromes / biohermes	B, R, F	bivalve (Lithiotis) 4, gastropods 2, corals 2, brachiopods 2, bioclasts 3, peloids 3	bouquet-like bioconstruction, slightly transported bioclasts	Open marine (inner–middle ramp)
2b	Diverse fauna facies	P, W-P	Echinoderms 2-3, bivalve 2-3, gastropods 2-3, brachiopods 2, bioclasts 3, lithoclasts (glauconite, quartz) 2-3	metric claystone intervals incised by P, W-P beds	Open marine (proximal middle ramp)
2c	Hummocky cross stratification facies	fs, slt, minor: ms	Lithoclastes (glauconite, quartz) 4, plant debris 1, bioclastes 1-2	Hummocky cross stratification	Open marine, storm-dominated (inner–middle ramp)
2d	Isolated coarse to fine storm deposits	fs, slt, cly	Lithoclastes (glauconite, quartz) 4, plant debris 2	wavy stratification	Open marine, storm-dominated (middle ramp)
3a	Marly facies	M-W, M, m minor: P, W	Ammonites 2, belemnites 2, echinoderms 2, oysters 2, gastropods 1-2, foraminifera 1-2, lithoclasts 0-2 (glauconie, quartz)	limestone/marl alternations, Zoophycos, Diplocraterion, Arenico-lites, Thalassinoides, Chondrites	Hemipelagic (outer ramp)
3b	Slumps/Turbidites	fs, slt, cly	Lithoclasts (glauconite, quartz) 1, intraclasts 1, plant debris 2, wood debris 2, bioclasts 2, ammonites 0-2, ophiures 0-1	turbidite sequences, flute casts, groove marks, linguoid ripples	Hemipelagic (proximal outer ramp)
3c	Muddy, argilaceous facies	slt, cly	Lithoclastes (glauconite, quartz) 4, plant debris 2-3, wood debris 2-3, bioclastes 1-2, ammonites 0-1, foraminifera 0-1	claystone-dominated facies interrupted by siltstone beds	Hemipelagic (distal outer ramp)

Fig. S2: Facies classification and interpretation. Numbers indicate the relative abundance of allochems: 0 = absent, 1 = present, 2 = frequent, 3 = abundant, 4 = dominant. Carbonate rock texture: m = marl, M = mudstone, W = wackestone, P = Packstone, G = grainstone, F = float-stone, R = rudstone, B = boundstone. Siliciclastic rock texture: cly = claystone, slt = siltstone, fs = fine sandstone, ms = medium sandstone, cs = coarse sandstone, vcs = very coarse sandstone, g = gravel.
